# Superficial incisional surgical site infections experimentally induced by *Staphylococcus aureus* in mice: the effect of *Bdellovibrio bacteriovorus* containing dressing*

**DOI:** 10.1007/s11274-025-04453-0

**Published:** 2025-06-25

**Authors:** Gülseren Maraş Baydoğan, Özlem Ceyhan, Pınar Sağıroğlu, Mustafa Ermiş

**Affiliations:** 1 Institute of Health Sciences, Surgical Nursing, Erciyes University, Kayseri, Türkiye; 2 Faculty of Health Sciences / Internal Medicine Nursing, Erciyes University, Kayseri, Türkiye; 3 Faculty of Medicine, Basic Medical Sciences, Medical Microbiology, Erciyes University, Kayseri, Türkiye; 4 Experimental Research Application and Research Center, Erciyes University, Kayseri, Türkiye

**Keywords:** Antimicrobial resistance, *Bdellovibrio bacteriovorus*, Surgical site infection, *Staphylococcus aureus*

## Abstract

*Bdellovibrio bacteriovorus* is an agent that stands out with its predatory properties and has recently been used against pathogens that are frequently resistant to antibiotics. The study was conducted experimentally to determine the effect of dressing application containing *Bdellovibrio bacteriovorus* on superficial incisional surgical site infection caused by *Staphylococcus aureus* in mice. In the study, mice were divided into 6 different groups, BB: *B. bacteriovorus*; NC: Negative Control; PC: Positive Control Methicillin Resistant *S. aureus*; MRSA + BB: Methicillin Resistant *S. aureus* + *B. bacteriovorus* dressing; MRSA + V: Methicillin Resistant *S. aureus* + Vancomycin; MRSA + BB + V: Methicillin Resistant *S. aureus* + *B. bacteriovorus* dressing + Vancomycin group. The treatment procedures were applied over a period of 3 days. Infection symptoms were monitored and recorded at the 24th, 48th, and 72nd hours. In the *Staphylococcus aureus* + Vancomycin group, all mice developed edema, redness, and fever at 24 h. At 48 h, all mice exhibited edema and redness, with 50% showing fever. At 72 h, 70% of the mice showed edema and redness, and 10% showed fever. In the *Staphylococcus aureus + Bdellovibrio bacteriovorus* + Vancomycin combined treatment group, all mice exhibited edema, redness, and fever at 24 h. At 48 h, only 20% of the mice showed redness. At 72 h, no edema, redness, fever, purulent discharge, or suture dehiscence was observed. Sepsis developed in 2 of 10 mice in the *Staphylococcus aureus + Bdellovibrio bacteriovorus* + Vancomycin group. The most effective treatment was in the *Staphylococcus aureus* + *Bdellovibrio bacteriovorus* + Vancomycin group. It was determined that sepsis findings were the least in the *Staphylococcus aureus + Bdellovibrio bacteriovorus* + Vancomycin group. *B. bacteriovorus* holds the potential to be an effective control agent in preventing or slowing resistance development.

## Graphical Abstract

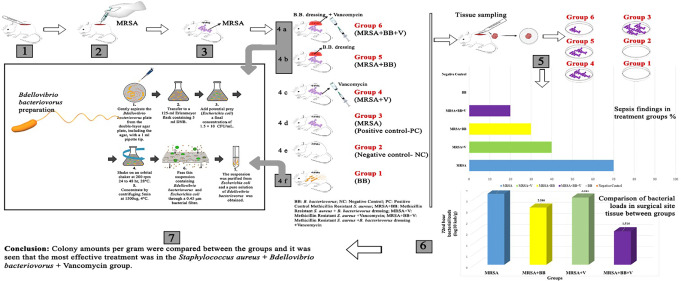

## Introduction

A Surgical Site Infection (SSI) is an infection that develops following a surgical procedure and emerges 30 or 90 days after the surgery. SSIs are categorized into three types: superficial, deep incisional, and organ/space SSIs. Superficial incisional SSI develops within 30 days after surgery and involves only the skin and subcutaneous tissues at the incision site (CDC 2016). SSI continues to be a significant global issue, even with advanced surgical procedures, asepsis, antisepsis, sterilization measures, and antibiotic prophylaxis. This situation leads to the use of more antibiotics, the development of antibiotic resistance, increased costs, morbidity and mortality. SSIs, which are an important clinical burden, also cause socioeconomic problems by causing hospital readmissions, long-term treatments, frequent surgeries and disabilities (Mezemir et al. 2020).

 According to the Centers for Disease Control and Prevention (CDC) report on healthcare-associated SSIs, it was reported that approximately 110,800 hospitalized patients developed infections in 2015. Approximately 14–16% of healthcare-associated infections are due to surgical site infections. According to the 2022 data from the National Healthcare Safety Network (NHSN), there was an approximate 4% increase in the standardized infection ratio for SSIs compared to the previous year (CDC 2024). The incidence of SSI varies between countries and hospitals. In developed nations like the United States of America, the United Kingdom, and Sweden, it runs between 2 and 6.4%. In developing countries such as India, Pakistan, Nepal, and Iran, the percentage is between 5.5 and 25% (Haque et al. 2021). Antibiotic-resistant bacteria not only contribute to an additional annual economic burden of $55–70 billion but are also responsible for at least 2 million illnesses and 23,000 deaths each year (Li and Webster 2018). Türkiye’s total Healthcare-Associated Infections (HAI) rate is 0.72%, as indicated by the statistics generated by Türkiye’s National Healthcare-Associated Infections Surveillance Network (NHSASN) in 2017. 8,194 of the 617,745 HAIs that were reported to the NHSIESA in 2017 were related to HAIs. The data from Turkish in 2017 shows that more than 1.0 SSI rate was observed in 25 out of 60 types of procedures. This highlights the ongoing significance of SSI in Türkiye (Ministry of Health 2018). 

The primary cause of surgical site infections (SSIs) is approximately 70–95% attributable to the patient’s endogenous flora. Among the most common bacteria are *Staphylococcus aureus* (*S. aureus*), coagulase-negative staphylococci, and *Escherichia coli* (*E. coli*) (Seidelman et al. 2023). In a cohort study involving 32 state hospitals, 24% of surgical site infections were associated with *S. aureus* (Seidelman 2022). A systematic review covering five European countries analyzed data from 178,902 patients who underwent surgery. The study found that 764 of these patients had S. aureus-related surgical site infections, representing an overall incidence of 0.4%. Of these infections, 46.3% were classified as superficial incisional surgical site infections. It was reported that 14% of the infections were caused by methicillin-resistant *S. aureus* (MRSA) (Mellinghoff et al. 2023). In Türkiye, it has been reported that 32.3% of SSIs are caused by Gram-positive cocci, with *S. aureus* being the most frequent etiological agent (Ministry of Health 2021). Recent trends indicate a rapid global increase in antibiotic resistance, which is expected to have increasingly adverse effects on global health, economics, and trade in the coming years. From a public health perspective, it is estimated that antibiotic resistance currently results in approximately 700,000 deaths annually. Furthermore, if the rate of resistance continues to rise at its current pace, it is projected that by 2050, antibiotic resistance could lead to 10 million deaths each year (Lee 2015; Frieri et al. 2017; WHO 2017). The rapid development of resistance to antimicrobial agents has necessitated the investigation of new agents and alternative mechanisms of action for the treatment of various bacterial infections (Yunusoğlu et al. 2020).

As a result, effective treatment options against antibiotic-resistant cases are seriously limited both in Türkiye and in the world. In light of this information, the use of various prebiotic and probiotic agents, both in combination with antibiotics and on their own, becomes an attractive option. Live and predatory bacteria, such as *Bdellovibrio bacteriovorus (B. bacteriovorus)*, are considered promising options for the treatment of antibiotic-resistant pathogens (Marine et al. 2020).

*Bdellovibrio bacteriovorus* is a live predatory bacterium that is effective on most Gram-negative bacteria (Milner et al. 2020). In studies examining the effect of *B. bacteriovorus* on Gram-negative bacteria, the suitability of its clinical use as a “live antimicrobial agent” has been stated (Patini et al. 2019; Saralegui et al. 2020; Maraş et al. 2023). *B.bacteriovorus* exhibits a unique biphasic life cycle consisting of an attack phase and a growth phase. During the attack phase, the motile predator locates and attaches to susceptible Gram-negative bacterial cells. Once attached, it penetrates the outer membrane and enters the periplasmic space, where it forms a structure known as a bdelloplast. Inside this protected environment, *B. bacteriovorus* digests the host cell’s contents and replicates. After consuming the host from within, it lyses the cell and releases progeny to continue the predatory cycle (Sockett 2009; Lambert et al., 2003). This mechanism is non-lytic for the predator but lethal for the prey. Although traditionally known to target Gram-negative bacteria, recent studies have suggested that *B. bacteriovorus* may also exhibit activity against Gram-positive bacteria, potentially via indirect mechanisms or biofilm disruption (Im et al. 2017; Dashiff & Kadouri, 2011). Understanding the specifics of this predatory process is essential to evaluating its potential as a live therapeutic agent, especially in infections caused by multidrug-resistant organisms (Negus et al. 2017). In a study conducted to investigate how *B. bacteriovorus*, a predator of Gram-negative bacteria, interacts with Gram-positive bacteria, it was found that the bacterium also has an effect on Gram-positive strains. For example, it was able to reduce the population of *S.aureus*, a common human pathogen, by 59%. These findings suggest that *B. bacteriovorus* is not only effective against Gram-negative bacteria and their biofilms, but may also interact with Gram-positive bacteria. However, the researchers emphasized that further studies are needed to better understand its effects on Gram-positive species (Im et al. 2018).

There are various studies on the effectiveness of *B. bacteriovorus* against antibiotic-resistant microorganisms; however, there are no studies on its use and efficacy as a dressing. Research examining the efficacy of live and predatory bacteria such as *B. bacteriovorus* on Gram-positive bacteria, and comparing it with standard methods, is needed. This study is the first in vivo investigation of the efficacy of *B. bacteriovorus* on Gram-positive bacteria-induced surgical site infections. This study was conducted as an experimental study to evaluate the effect of *B.* bacteriovorus-containing dressing application on superficial incisional surgical site infections induced by MRSA in mice.

## Material and method

### Research and ethical considerations

The research was conducted at Erciyes University Experimental Research Application and Research Center (DEKAM). Ethical approval was obtained from the Erciyes University Local Ethics Committee for Animal Experiments (approval no. 2021/239 dated 01.12.2021) and the Faculty of Health Sciences Academic Committee (approval no. 2021/30 dated 13.10.2021). Institutional permission was also granted by the Experimental Research Application and Research Center (approval no. 169480 dated 22.12.2021).

### Experimental animals

A total of 70 Balb-c mice (8–10 weeks old, female, weighing 25–35 g) were used in the study. Mice were divided into 6 groups (Table 1). Considering the mice that may die as a result of surgical infection, the minimum sample number was 10 for each group, and the relevant parameters were calculated by Erciyes University Biostatistics Department using similar experimental studies (Maraş et al. 2023; Dhasmana at al., 2018; Chadha et al. 2017).

**Table 1:** Experimental groups

Groups	Implementation	Number of Mice
	Pre-study	10
Group 1 (BB)	*B. bacteriovorus*	10
Group 2 (negative control- NC)	Control group	10
Group 3 (MRSA) (positive control-PC)	*Staphylococcus aureus*	10
Group 4 (MRSA + V)	*Staphylococcus aureus* + Vancomycin (antibiotic)	10
Group5 (MRSA + BB)	*Staphylococcus aureus + B. bacteriovorus* dressing	10
Group 6 (MRSA + BB + V)	*Staphylococcus aureus + B. bacteriovorus* dressing + Vancomycin (antibiotic)	10
**Total**		70

### Bacterial isolates

#### Culture, application, and infection induction of MRSA strain

The MRSA strain used in the study was isolated from Erciyes University Medical Faculty Hospital and considered a pathogenic agent. The isolated *S. aureus* colonies were passaged and incubated at 37 °C for 18–24 h. Following incubation, the colonies were collected using an inoculation loop, and bacterial suspensions of various concentrations (1.5 × 10^6^, 1.5 × 10^7^, and 1.5 × 10^8^ CFU/mL) were prepared using a spectrophotometric method. The minimum MRSA inoculum dose that resulted in bacterial growth at the wound site without causing mortality in the mice was determined to be 1.5 × 10^7^ CFU/mL. The MRSA bacterial suspension was injected into the wound site and the bilateral wound edges using an insulin injector, with a volume of 0.5 mL administered through the creation of a surgical incision.

#### *Bdellovibrio bacteriovorus* strain

**Fig. 1:**
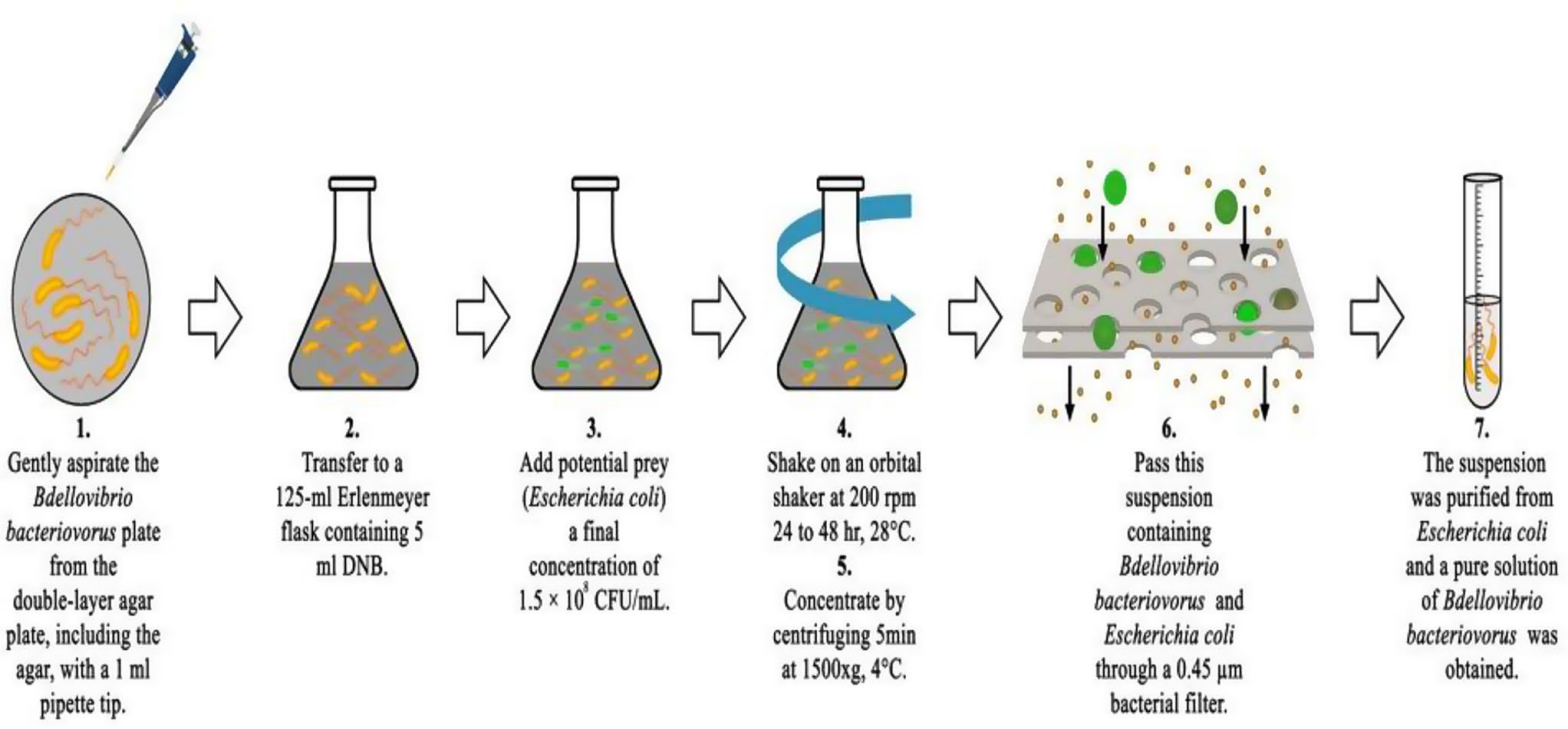
*Bdellovibrio bacteriovorus* preparation diagram

The *B. bacteriovorus* strain was obtained from Prof. Dr. Edouard Jurkevitch at the Hebrew University and the cryotube containing *B. bacteriovorus* HD-100 and *E. coli* was stored at -80 °C. The predatory bacteria were prepared according to the *B. bacteriovorus* protocol developed by Jurkevitch et al. (Figure I). In a 125 mL Erlenmeyer flask containing HM buffer, both the prey (*E. coli*) and the predator (*B. bacteriovorus*) were added. The bacterial suspension was incubated at 28 °C with shaking at 200 rpm for 24–48 h, reaching a final predator concentration of 1.5 × 10^8^ CFU/mL. The suspension containing *B. bacteriovorus* and *E. coli* was filtered through a 0.45 μm bacterial filter. This process separated the bacterial suspension from E. coli, resulting in a pure *B. bacteriovorus* solution (Fig. 1) (Jurkevitch et al. 2000). The treatment dose of B. bacteriovorus used in the studies, aimed at eliminating MRSA infections in the wound area, was established at 1.5 × 10^8^ CFU/ mL. (Baker et al. 2017; Im et al. 2017; Maraş et al. 2023).

### In vivo study

The treatment procedures outlined in Table 2 were applied over a period of 3 days. Following a 12-hour fasting period, the mice were anesthetized with intraperitoneal injections of 50 mg/kg Ketamine and 10 mg/kg Xylazine. The mice’s dorsal hairs were shaved using an electric razor before the incision, and the incision area was cleaned with povidone-iodine. A full-thickness wound model was created on the mice’s backs in the regio interscapularis caudal area, extending to the deep fascia but not including it, with a length of 2 cm. Based on preliminary studies, the MRSA infection dose (1.5 × 10^7^ CFU/mL) was injected as 0.5 mL into both the wound site and the bilateral wound edges using an insulin syringe. Additionally, suture threads were impregnated in bacterial suspensions of the same concentration for one minute. The bacterial solution was allowed to absorb into the wound site for 5 min. After inoculation, the incision area was closed with 3/0 silk sutures to create an incisional wound model.

**Table 2:** Work flowchart

Groups	0th hour	24th hour	48th hour	72nd hour	
Group 1 (BB)	Surgical incision + *B.bacteriovorus* inoculation	Recording of infection symptoms	Recording of infection symptoms	Recording of infection symptoms	Recording of infection symptoms + Tissue culture + Sacrifice + Organ dissection
Group 2 (Negative control- NC)	Surgical incision	Recording of infection symptoms	Recording of infection symptoms	Recording of infection symptoms	
Group 3 (MRSA) (Pozitif control-PK**)**	Surgical incision + *MRSA* inoculation	Recording of infection symptoms	Recording of infection symptoms	Recording of infection symptoms	
Group 4 (MRSA + V)	Surgical incision + *MRSA* inoculation	2 × 110 mg/kg intraperitoneally Vancomycin (antibiotic) + Recording of infection symptoms	2 × 110 mg/kg intraperitoneally Vancomycin (antibiotic) + Recording of infection symptoms	2 × 110 mg/kg intraperitoneally Vancomycin (antibiotic) + Recording of infection symptoms	
Group 5 (MRSA + BB)	Surgical incision + *MRSA* inoculation	*B.bacteriovorus* dressing impregnated with Wattman filter paper 3 × 1 + Recording of infection symptoms	*B.bacteriovorus* dressing impregnated with Wattman filter paper 3 × 1 + Recording of infection symptoms	*B.bacteriovorus* dressing impregnated with Wattman filter paper 3 × 1 + Recording of infection symptoms	
Group 6 (MRSA + BB + V)	Surgical incision + *MRSA* inoculation	*B.bacteriovorus* dressing impregnated with Wattman filter paper 3 × 1 + 2 × 110 mg/kg intraperitoneally Vancomycin (antibiotic) + Recording of infection symptoms	*B.bacteriovorus* dressing impregnated with Wattman filter paper 3 × 1 + 2 × 110 mg/kg intraperitoneally Vancomycin (antibiotic) + Recording of infection symptoms	*B.bacteriovorus* dressing impregnated with Wattman filter paper 3 × 1 + 2 × 110 mg/kg intraperitoneally Vancomycin (antibiotic) + Recording of infection symptoms	

BB: *B. bacteriovorus*; NC: Negative Control; PC: Positive Control Methicillin Resistant *S. aureus;* MRSA + BB: Methicillin Resistant *S. aureus + B. bacteriovorus* dressing; MRSA + V: Methicillin Resistant *S. aureus* + Vancomycin; MRSA + BB + V: *Methicillin Resistant S. aureus* + *B. bacteriovorus* dressing *+* Vancomycin

In the negative control (NC) group, an incision was made but no bacterial infection was introduced, and no treatment was applied. This group was left to heal naturally and served to control for the inflammatory effects caused solely by the surgical incision, effectively functioning as a sham surgery group.

In the *B. bacteriovorus* dressing groups, 0.5 mL of *B. bacteriovorus* (1.5 × 10^8^ CFU/mL) impregnated in Wattman filter paper was used for dressing three times daily. In the antibiotic groups, 2 × 10^10^ mg/kg Vancomycin (antibiotic) was administered intraperitoneally. Mice were individually housed with drinking water containing 100–300 mg/kg Paracetamol (suspension). Observations for edema, redness, purulent discharge, suture dehiscence, and fever were recorded daily on a control list. In the *B. bacteriovorus* dressing groups, the dressing procedure was discontinued 18 h before sacrifice to prevent contamination. On the 72nd hour, 50 mg/kg Ketamine and 10 mg/kg Xylazine were administered intraperitoneally, followed by cervical dislocation for sacrifice.

### Antibiotic treatment

Vancomycin, used in treating surgical site infections caused by *S. aureus* in Türkiye, was used as an antibiotic in this study. The dose of vancomycin was administered 2 × 110 mg/kg intraperitoneally (Yee et al. 2022).

### Microbiological examination

After the mice were sacrificed, 1 gram of tissue was homogenized in saline after measuring with a precision scale from the incision site infection area. Serial dilutions (10⁻¹, 10⁻², 10⁻³ … 10⁻⁸) of the homogenized sample were inoculated onto Sheep Blood Agar and incubated at 37 °C for 18–24 h. Quantitative colony counting was performed, and results were expressed as CFU/g. The bacteria showing growth were confirmed with the automated identification and antibiogram device (BD Phoenix). Colonies were expressed as CFU, and the quantitative culture results were recorded as Log10 CFU/g (Chadha et al. 2016; Huang et al. 2020; Mcdaniel and Allen 2019; Maraş et al. 2023). The sacrificed mice’s heart, liver, lung and spleen tissues were inoculated on Sheep Blood Agar to check whether sepsis developed. Growth results were recorded after incubation at 37^0^C for 18–24 h.

### Data evaluation

The data were analyzed using SPSS version 21.0 (Statistical Package for the Social Sciences, Chicago, IL, USA). Descriptive statistics were presented as percentages (%) and mean ± standard deviation (x̄ ± SD). Bacterial colony counts were converted to logarithmic values (Log₁₀) prior to analysis. The assumptions of one-way ANOVA were tested before conducting the analysis. The Shapiro–Wilk test was used to assess the normality of the data distribution, and the Levene’s test was applied to verify the homogeneity of variances. Both assumptions were met (*p* > 0.05). Following this, group comparisons were conducted using one-way ANOVA, and post hoc Tukey’s HSD test was used to determine pairwise differences. The statistical significance level was set at *p* < 0.05 with a 95% confidence interval. Additionally, linear regression analysis was performed to identify significant variables predicting the dependent variable. The model evaluated the impact of treatment groups on colony counts. The standardized regression coefficient (β) was used to compare the relative effects of treatment groups on the dependent variable (Field 2018).

## Results

The study’s findings to evaluate the effect of *Bdellovibrio bacteriovorus*-containing dressing application on superficial incisional surgical site infections caused by methicillin-resistant *Staphylococcus aureus* in mice are given below.

### Signs and symptoms of infection

**Fig. 2:**
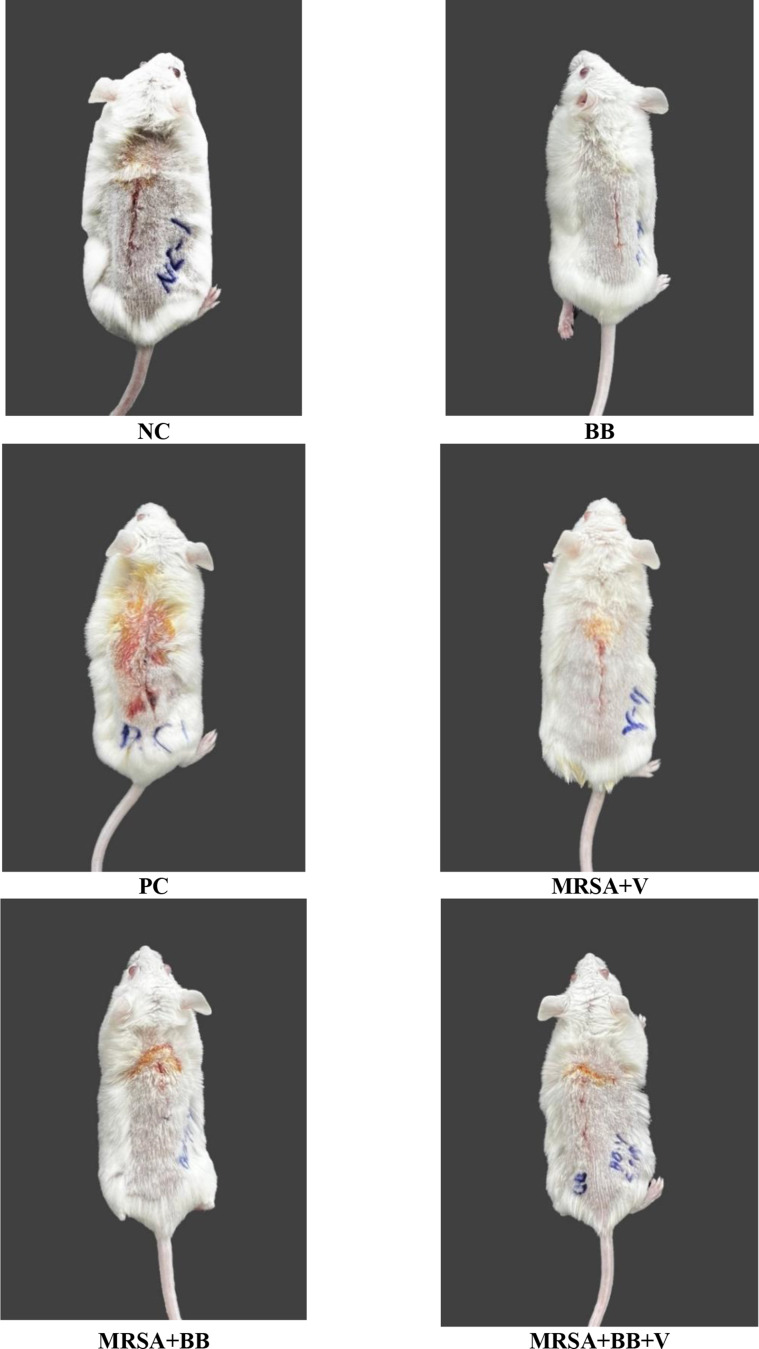
Surgical site incision images of treatment groups. BB: *B. bacteriovorus*; NC: Negative Control; PC: Positive Control Methicillin Resistant *S. aureus;* MRSA + BB: Methicillin Resistant *S. aureus + B. bacteriovorus* dressing; MRSA + V: Methicillin Resistant *S. aureus* + Vancomycin; MRSA + BB + V: *Methicillin Resistant S. aureus* + *B. bacteriovorus* dressing *+* Vancomycin

**Fig. 3:**
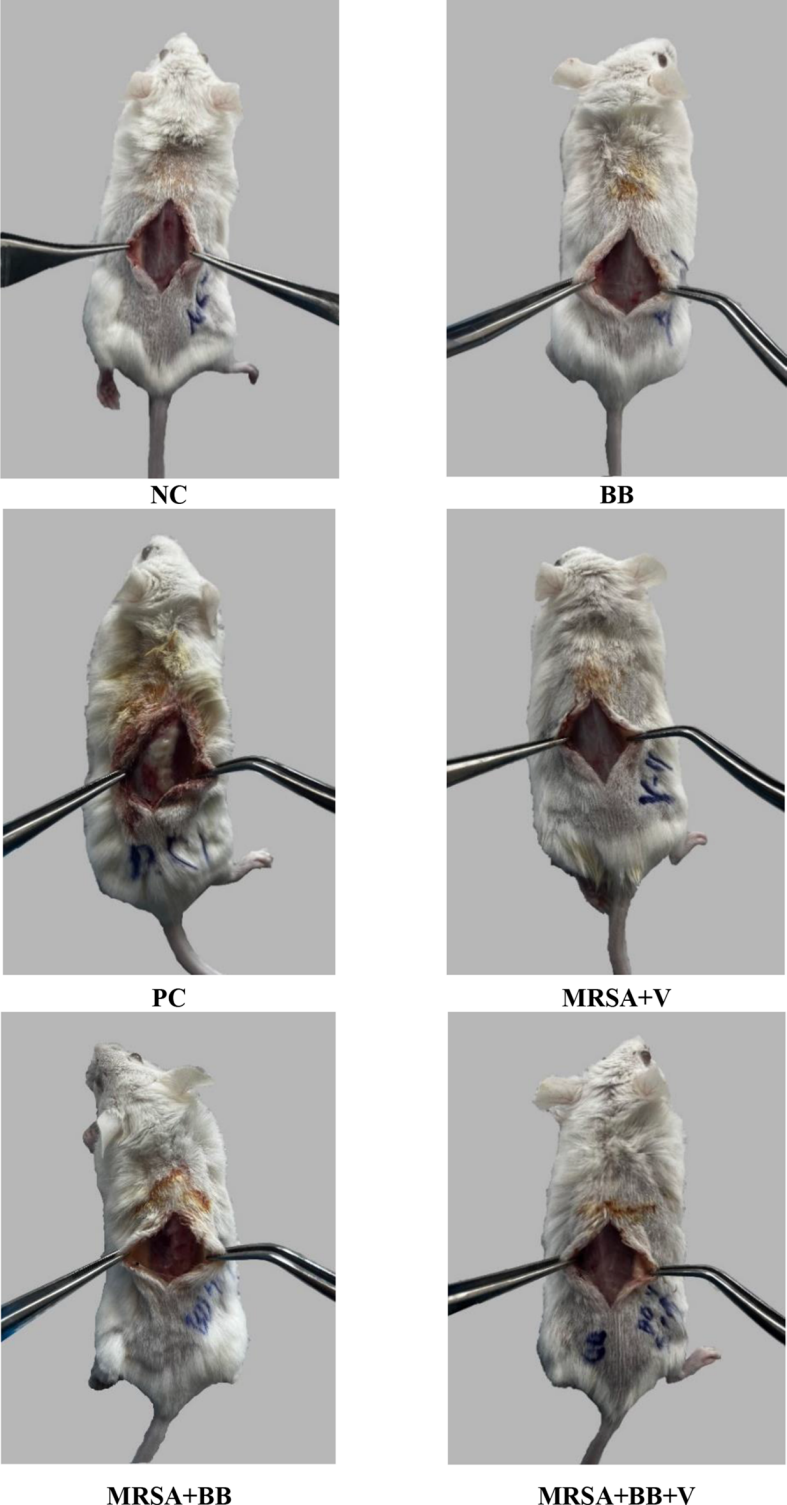
Surgical site tissue images of treatment groups. BB: *B. bacteriovorus*; NC: Negative Control; PC: Positive Control Methicillin Resistant *S. aureus;* MRSA + BB: Methicillin Resistant *S. aureus + B. bacteriovorus* dressing; MRSA + V: Methicillin Resistant *S. aureus* + Vancomycin; MRSA + BB + V: *Methicillin Resistant S. aureus* + *B. bacteriovorus* dressing *+* Vancomycin

The infection symptoms of the negative control, positive control, *B. bacteriovorus*, MRSA + BB, MRSA + V, and MRSA + BB + V groups were compared. The infection symptoms of the mouse groups at 24, 48, and 72 h are presented in Table 3. In the negative control group, all mice exhibited edema and redness at 24 h. At 48 and 72 h, no edema, redness, or fever was observed. In the positive control group, all mice showed edema and redness at 24 and 48 h, and at 72 h, all mice continued to show edema and redness, with 80% also exhibiting fever. In the *B. bacteriovorus* group, all mice displayed edema and redness at 24 h, while at 48 h, 30% showed edema and redness, and at 72 h, no mice exhibited these symptoms. In the MRSA + BB group, all mice showed edema and redness at 24 h, but no edema, redness, or fever was observed at 48 and 72 h. In the MRSA + V group, all mice developed edema, redness, and fever at 24 h. At 48 h, all mice exhibited edema and redness, with 50% showing fever. At 72 h, 70% of the mice showed edema and redness, and 10% showed fever. In the MRSA + BB + V combined treatment group, all mice exhibited edema, redness, and fever at 24 h. At 48 h, only 20% of the mice showed redness. At 72 h, no edema, redness, fever, purulent discharge, or suture dehiscence was observed. No purulent discharge or suture dehiscence was observed in any of the groups at 24, 48, and 72 h (Figs. 2 and 3).

**Table 3:** Symptoms and signs of infection in the groups

**Groups**	**Edema**	**Redness**	**Purulent** **discharge**	**Suture** **dehiscence**	**Ateş Fever**
	**24th hour** **Number of mice/number of symptoms**				
BB	10/10	10/10	10/0	10/0	10/0
NC	10/10	10/10	10/0	10/0	10/0
PC	10/10	10/10	10/0	10/0	10/10
MRSA + BB	10/10	10/10	10/0	10/0	10/0
MRSA + V	10/10	10/10	10/0	10/0	10/10
MRSA + BB + V	10/10	10/10	10/0	10/0	10/10
	**48th hour** **Number of mice / number of symptoms**				
BB	10/3	10/3	10/0	10/0	10/0
NC	10/0	10/0	10/0	10/0	10/0
PC	10/10	10/10	10/0	10/0	10/10
MRSA + BB	10/0	10/0	10/0	10/0	10/0
MRSA + V	10/10	10/10	10/0	10/0	10/5
MRSA + BB + V	10/0	10/2	10/0	10/0	10/0
	**72nd hour** **Number of mice/number of symptoms**				
BB	10/0	10/0	10/0	10/0	10/0
NC	10/0	10/0	10/0	10/0	10/0
PC	10/10	10/10	10/0	10/0	10/8
MRSA + BB	10/0	10/0	10/0	10/0	10/0
MRSA + V	10/7	10/7	10/0	10/0	10/1
MRSA + BB + V	10/0	10/0	10/0	10/0	10/0

BB: *B. bacteriovorus*; NC: Negative Control; PC: Positive Control Methicillin Resistant *S. aureus;* MRSA + BB: Methicillin Resistant *S. aureus + B. bacteriovorus* dressing; MRSA + V: Methicillin Resistant *S. aureus* + Vancomycin; MRSA + BB + V: *Methicillin Resistant S. aureus* + *B. bacteriovorus* dressing *+* Vancomycin

### Signs of sepsis

**Fig. 4:**
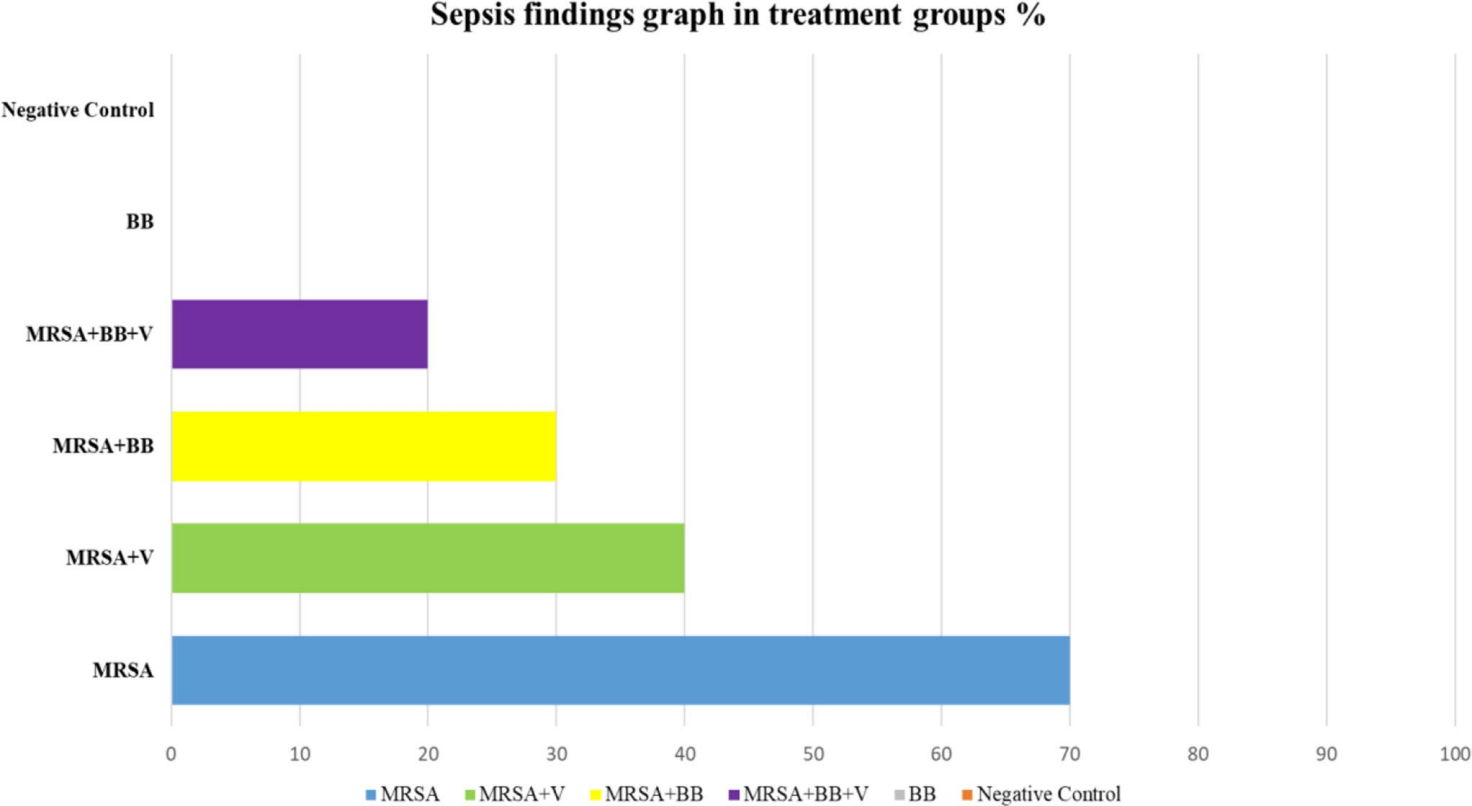
Sepsis findings graph in treatment groups. BB: *B. bacteriovorus*; NC: Negative Control; PC: Positive Control Methicillin Resistant *S. aureus;* MRSA + BB: Methicillin Resistant *S. aureus + B. bacteriovorus* dressing; MRSA + V: Methicillin Resistant *S. aureus* + Vancomycin; MRSA + BB + V: *Methicillin Resistant S. aureus* + *B. bacteriovorus* dressing *+* Vancomycin

When the development of sepsis in the groups is analyzed in Table 4; Fig. 4, no sepsis was observed in the BB and negative control groups after examination of the liver, spleen, heart, and lung tissues of the mice after sacrifice at 72 h. Sepsis developed in 7 of 10 mice in the positive control group, 4 of 10 in the MRSA + V group, 3 of 10 in the MRSA + BB group, and 2 of 10 mice in the MRSA + BB + V group.

**Table 4:** Sepsis findings in treatment groups

BB *n* (%)	NC *n* (%)	PC *n* (%)	MRSA + V *n* (%)	MRSA + BB *n* (%)	MRSA + BB + V *n* (%)
0 (%0)	0 (0%)	7 (%70)	4 (%40)	3 (%30)	2 (%20)

### Bacteria loads

**Fig. 5:**
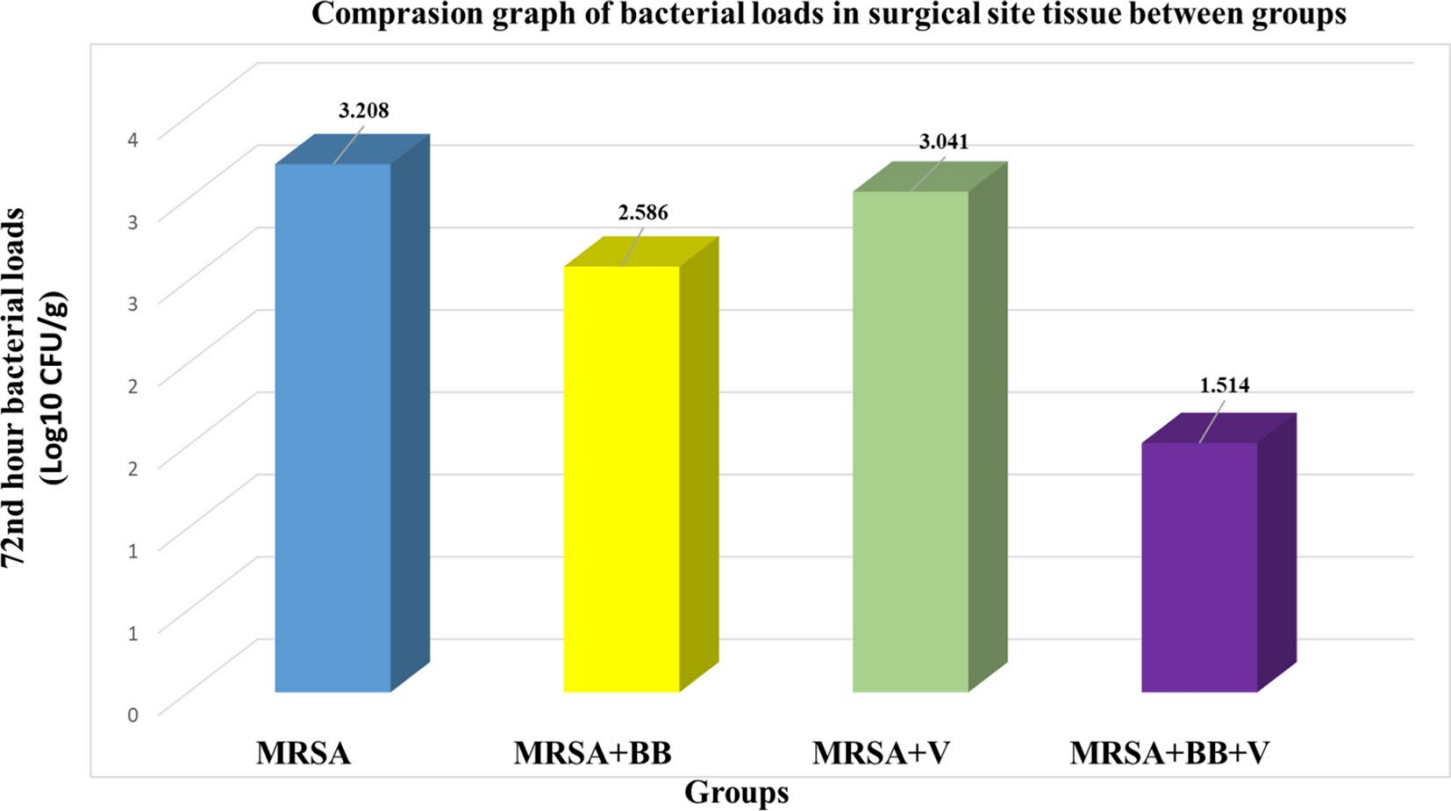
Comparison graph of bacterial loads in surgical site tissue between groups. BB: *B. bacteriovorus*; NC: Negative Control; PC: Positive Control Methicillin Resistant *S. aureus;* MRSA + BB: Methicillin Resistant *S. aureus + B. bacteriovorus* dressing; MRSA + V: Methicillin Resistant *S. aureus* + Vancomycin; MRSA + BB + V: *Methicillin Resistant S. aureus* + *B. bacteriovorus* dressing *+* Vancomycin

At the conclusion of the study, the colony counts and bacterial loads per gram of surgical site tissue were compared across the groups (Table 5, and Fig. 5). The highest bacterial loads per gram of wound tissue were observed in the positive control (MRSA), MRSA + V, MRSA + BB, and MRSA + BB + V groups, respectively. The average colony count for the positive control group was 3.208 ± 0.180 CFU/g, for the MRSA + BB group it was 2.586 ± 0.570 CFU/g, for the MRSA + V group it was 3.041 ± 0.323 CFU/g, and for the MRSA + BB + V group it was 1.514 ± 0.533 CFU/g. Based on the average colony counts, the positive control group had the highest average colony count. When comparing bacterial loads, the MRSA + BB + V and MRSA + BB groups had significantly lower bacterial loads compared to the positive control (MRSA) group (*p* < 0.001). No statistically significant difference was observed between the positive control (MRSA) and MRSA + V groups, nor between the MRSA + V and MRSA + BB groups (*p* > 0.05). The most effective treatment in reducing bacterial load per gram was found to be the combined application of *B. bacteriovorus* dressing and antibiotic in the MRSA + BB + V group. Subsequently, the MRSA + BB and MRSA + V treatment groups were observed to be effective, in that order.

**Table 5:** Comparison of bacterial loads in surgical site tissue between groups

Groups	72nd hour mean ± standard deviation (Log10 CFU/g)	Test *p*
MRSA + V MRSA + BB MRSA + BB + V MRSA (pozitive control**)**	3.041 ± 0.323^b, c^ 2.586 ± 0.570^b^ 1.514 ± 0.533^a^ 3.208 ± 0.180^c^	**F:31.119** *p* < 0.001*

BB: *B. bacteriovorus*; NC: Negative Control; PC: Positive Control Methicillin Resistant *S. aureus;* MRSA + BB: Methicillin Resistant *S. aureus + B. bacteriovorus* dressing; MRSA + V: Methicillin Resistant *S. aureus* + Vancomycin; MRSA + BB + V: *Methicillin Resistant S. aureus* + *B. bacteriovorus* dressing *+* Vancomycin F: ANOVA; *P* < 0.001 * One-way ANOVA and post-hoc Tukey HSD analysis was conducted. The same superscripts indicate no difference between groups, while different superscripts indicate a difference between groups

The linear regression analysis in Table 6 examined the relationship between colony number and treatment groups. The model obtained explains 75.4% of the total variance of the change in colony count. The MRSA + BB + V treatment group was the best predictor of the decrease in colony count.

**Table 6:** The relationship between bacterial loads and treatment groups: multiple linear regression analysis

Independent Variables	B†	SE†	β	*p*	95% CI	VIF	Tolerance
MRSA + V	0.475	0.193	0.852	**0.010**	0.160–0.790	1.301	0.769
MRSA + BB	0.183	0.193	0.579	**0.032**	0.021–0.345	1.065	0.939
MRSA + BB + V	-0.069	0.193	-0.203	0.408	-0.257–0.120	1.268	0.789
MRSA (Reference)	1.394	0.430		**0.018**	0.342–2.477	-	
R² (Adjusted): 0.754* DW:2.606 Model p: 0.029*							

B†: unstandardized regression coefficient; SE: standard error; β: standardized regression coefficient; 95% CI: confidence interval; DW: Durbin-Watson; VIF: Variance Inflation Factor; Tolerance: 1/VIF; *R^2^: Adjusted R-squared *p < 0.05; BB: *B. bacteriovorus*; NC: Negative Control; PC: Positive Control Methicillin Resistant *S. aureus;* MRSA + BB: Methicillin Resistant *S. aureus + B. bacteriovorus* dressing; MRSA + V: Methicillin Resistant *S. aureus* + Vancomycin; MRSA + BB + V: *Methicillin Resistant S. aureus* + *B. bacteriovorus* dressing *+* Vancomycin

## Discussion

This study used a live organism *B. bacteriovorus* dressing to treat superficial incisional surgical site infections. According to the literature, in vitro, experiments have shown that *B. bacteriovorus* cannot prey on eukaryotic cells. As a result, it has not been demonstrated to directly endanger animal health (Monappa et al. 2014; Shatkes et al. 2015; Atterbury et al. 2016; Gupta et al. 2016; Romanowski et al. 2016; Raghunathan et al. 2019). Due to its diverse array of prey, this bacterium appears to possess the capacity to be a viable option for treating infections in living organisms. Throughout the investigation, this study found no systemic or local adverse effects caused by *B. bacteriovorus* in animals. The absence of any adverse side effects of *B. bacteriovorus* in mice is a positive indicator for future therapeutic trials.

In our superficial incisional surgical site infection model, the infection symptoms (edema, redness, purulent discharge, suture dehiscence, fever) in the mice were monitored at 24, 48, and 72 h. It was observed that MRSA + BB + V, MRSA + BB and MRSA + V groups had the least number of signs of infection at 72nd hours, respectively. Although it was thought that *B. bacteriovorus* dressing applied in combination with antibiotics in the study would result in less efficacy because *B.* Bacteriovorus is sensitive to antibiotics, MRSA + BB + V was the most effective group. When the infection symptoms of the mice in the group in which *B. Bacteriovorus* was injected alone without *MRSA* infection were examined, it was observed that *B. Bacteriovorus* facilitated natural wound healing. In a study evaluating tissue sections of mice with infected burn wounds, it was reported that on the 4th day after burn induction, an inflammatory response was observed. In the group treated with *B. bacteriovorus*, the burn rate decreased after the 14th day, while the control group exhibited a high inflammatory response. Additionally, the group treated with *B. bacteriovorus* showed higher fibroblastic migration and epithelialization compared to both the control and antibiotic-treated groups (Tajabadi et al. 2022). It is stated that in cases such as surgical trauma, infection, and burn, *B. Bacteriovorus* destroys pathogens that cause skin infections by stimulating the production of immune cells and may positively affect host health and skin healing (Ito et al. 2012). Liu et al. (2023) investigated the effects of *B.* bacteriovorus-containing hydrogel on the healing and antibacterial mechanisms of wound infection caused by *Vibrio* vulnificus. The study divided mice into Aquacel Ag (ionic silver-containing hydrogel), *B. bacteriovorus* containing hydrogel, and positive and negative control groups. The Aquacel Ag group showed significantly superior wound healing on day 3 compared to the control group with exudate infection. However, there was no statistical difference with the uninfected control group throughout the entire wound-healing process. In contrast, the hydrogel group containing *B. bacteriovorus* appeared to accelerate wound healing compared to the other groups, including the negative control group. Interestingly, the hydrogel group containing *B. bacteriovorus* improved the healing of infected wounds much better than non-infected ones. The good wound healing effect of the *B. bacteriovorus*-containing hydrogel group may be related to its antibacterial solid ability and its hydrogel structure.

In the literature, various studies have examined the efficacy of different applications in surgical site infections caused by Gram-positive-negative bacteria and MRSA. However, no study has investigated the efficacy of *B. bacteriovorus* in surgical site infections caused by Gram-positive bacteria and MRSA. MRSA is a multidrug-resistant “superbug” that is highly resistant to antibiotics such as penicillins, cephalosporins, chloramphenicol, and rifampicin, making clinical treatment extremely challenging. Additionally, MRSA infections are reported to be one of the world’s significant infectious diseases that seriously threaten human health, with high morbidity and mortality rates, drawing global medical community attention. Resistance to vancomycin, which has long been considered the best drug for treating MRSA infections, is increasing daily, causing widespread concern in the healthcare community (Guo et al. 2020; Cheung et al. 2021). With the increase in antibiotic resistance, new antibiotics have necessitated further research on existing ones and a review of new therapeutic strategies. Our application of *B. Bacteriovorus* nanotechnology is one of these new therapeutic strategies.

When the development of sepsis in the study groups was examined, no sepsis was observed in the *B. Bacteriovorus* and negative control group due to the examination of the mice’s liver, spleen, heart, and lung tissues after sacrifice at 72nd hours. The rate of sepsis was 70% in the positive control group, 40% in the MRSA + V group, 30% in the MRSA + BB group, and 20% in the MRSA + BB + V group. Bacterial sepsis is an important cause of death worldwide. *S. aureus* is among the pathogens most frequently associated with sepsis-related deaths. As a result of the alarming spread of antibiotic resistance, anti-virulence strategies are often recommended to treat sepsis caused by *S. aureus*. The efficacy of therapeutic interventions for sepsis is not feasible in the clinic and requires animal models. Our results revealed a critical role of bacterial virulence, especially MRSA, in the development of sepsis. In addition, this result suggests that the combined application of antibiotics and *B.* Bacteriovorus is effective in the group due to both systemic and local efficacy. Our results also support the idea that reducing the bacterial load early during infection plays a critical role in the development of sepsis.

When comparing bacterial loads, the MRSA + BB + V and MRSA + BB groups had significantly lower bacterial loads compared to the positive control (MRSA) group (*p* < 0.001). The most effective treatment in reducing bacterial load per gram was found to be the combined application of *B. bacteriovorus* dressing and antibiotic in the MRSA + BB + V group. The linear regression analysis model explained 75.4% of the total variance of the change in colony count. The MRSA + BB + V treatment group was the best predictor of the decrease in colony count. Subsequently, the MRSA + BB and MRSA + V treatment groups were observed to be effective, in that order. *B. bacteriovorus* is sensitive to some antibiotics and agents that specifically target the cell wall can inhibit its growth or motility. However, as observed in our study, despite the possible inhibitory effect of Vancomycin on *B. bacteriovorus*, the highest efficacy of the MRSA + BB + V group can be explained by several mechanisms: The antibiotic dose and timing of application were such that they did not inhibit the hunting activity of *B. bacteriovorus*, the effect of vancomycin increased by weakening MRSA, limited exposure to the antibiotic in some microenvironments, and the rapid effect of *B. bacteriovorus* starting before the antibiotic may have been effective in this result. *B. bacteriovorus* alone may have borderline characteristics on Gram-positive features (e.g., MRSA); however, it can be combined with an antibiotic (e.g., vancomycin), and both effects can complement each other (Cavallo et al. 2021). Studies such as Durán et al. (2007) have shown that the effectiveness of the predator bacteria against Gram-positives increases when distributed together with antibiotics. Marine et al. (2020) reported that *B. bacteriovorus* is resistant to certain antibiotics (e.g., trimethoprim) and sensitive to others. The antibiotic used in the study (e.g.,. vancomycin) may not have completely inhibited *B. bacteriovorus*, or the dosage and application method may have provided opportunities for the predator to maintain its resistance. In addition, Monnappa et al. (2013) reported that the predator prevents the spread of antibiotic resistance genes by breaking down the DNA of the bacteria it hunts; this situation and the solution are known to limit the prolongation of resistance.

Sun et al. (2017) conducted a study on the predation efficacy of *B. bacteriovorus* on *Klebsiella pneumoniae* (*K. pneumoniae*) and *E. coli*. They reported a decrease of 83.4% in *E. coli* and 81.8% in *K. pneumoniae* after 48 h in biofilm plaques. Consequently, it was determined that *B. bacteriovorus* exhibits a highly potent predatory activity. The research conducted by Baker et al. (2017) investigated the capacity of *B. bacteriovorus* to decrease the population of *K. pneumoniae* cells in human serum. The study demonstrated that the predatory bacteria have a significant ability to control the disease. The study demonstrated that *B. bacteriovorus* can effectively preying on human serum from the clinical isolate *K. pneumoniae*, a significant contributor to carbapenem-resistant infections. In another study, it was reported that *B. bacteriovorus* administered intranasally in an experimental pneumonia model in which *K.* pneumoniae was used as an infectious agent could reduce the *K. pneumoniae* bacterial load in lung tissue by more than 3.0 log10 on average and this decrease was statistically significant [40]. Tajabadi et al. (2022) conducted a study on using *B. bacteriovorus* to treat burn wound infection caused by *Pseudomonas aeruginosa* in mice. The administration of Meropenem and *B. bacteriovorus* decreased the severity of the wound infection compared to the control group. While the control and antibiotic groups showed an increase in bacterial count, no such rise was detected in the *B. bacteriovorus* group. This study demonstrated that *B. bacteriovorus* exhibited superior efficacy compared to antibiotics in the treatment of burn wound infection in mice, particularly when delivered promptly following bacterial exposure. In a study evaluating the ability of *B. bacteriovorus* HD100 strain to prevent *P. aeruginosa* proliferation in a bacterial keratitis model in rabbits, signs of ocular inflammation were evaluated 24 h later according to the McDonald-Shadduck grading system, and *P. aeruginosa* proliferation was reduced 7-fold in eyes treated with *B. bacteriovorus*. *B. bacteriovorus* injected after *P.* aeruginosa showed a significant reduction in *P. aeruginosa* proliferation (1.03 × 10^4^ CFU at 24 h, *p* < 0.01). This reduction did not significantly affect the overall corneal score, but was associated with a significant reduction in the frequency of corneal perforations (4%, *p* < 0.001) (De Souza et al. 2023).

## Conclusions and recommendations

Based on literature reviews, this experimental study is the first to apply *B. bacteriovorus* in a mouse model of superficial incisional surgical site infections caused by *S. aureus*. The study demonstrated that the predatory *B. bacteriovorus* could be effective in treating surgical site infections when used locally, both alone and in combination with standard treatments, against Gram-positive bacteria. At the end of the study, no signs of infection were detected at 72 h in the MRSA + BB and MRSA + BB + V groups. The MRSA + BB + V group exhibited the fewest sepsis symptoms, indicating the combined dressing and antibiotic treatment’s effectiveness. The MRSA + BB + V group was found to be the most effective in reducing the bacterial load per gram in the treatment groups, and it was identified as the best predictor for colony count reduction.

The rapid development of resistance to antimicrobial agents suggests an increasing need for new agents and different mechanisms of action to treat various bacterial infections. Predictions indicate that antibiotic resistance will have significant global impacts in the coming years. In this context, *B. bacteriovorus* holds the potential to be an effective control agent in preventing or slowing resistance development. Comprehensive studies on the inhibition potential of B. bacteriovorus, its interactions with the immune system, and its potential use against deep tissue and systemic infections caused by resistant pathogens are warranted. This study represents the first step towards the therapeutic application of *B. bacteriovorus* for other animals and potentially human infections. Further research is recommended to evaluate its potential as both a probiotic and antibiotic for human and animal health. As highlighted in this study, B. bacteriovorus could be a promising “living antibiotic” for combating antibiotic-resistant pathogens in the future.

## Data Availability

No datasets were generated or analysed during the current study.

## Abbreviations

BB: *Bdellovibrio bacteriovorus*
CDC: Centers for Disease Control and Prevention
CFU/mL: Coloni-forming units/ milliliter
E. coli: *Escherichia coli*
HAI: Healthcare-associated infections
MRSA: Methicillin-resistant Staphylococcus aureus
NC: Negative control
NHSASN: Türkiye’s National Healthcare-Associated Infections Surveillance Network
NNIS: National Nosocomial Infection Surveillance System
PC: Positive Control
*S. aureus*: *Staphylococcus aureus*
SSI: Surgical site infection
V: Vancomycin
Β: Beta
